# Fostering Physician‐Patient Partnerships: The Importance of Embracing the Ontological and Epistemological Understandings of Knowledge

**DOI:** 10.1111/hex.70484

**Published:** 2025-11-21

**Authors:** Nicolas Fernandez, Joachim P. Sturmberg

**Affiliations:** ^1^ Faculty of Medicine, Department of Family Medicine and Emergency Medicine Université de Montréal Montréal Canada; ^2^ College of Health, Medicine and Wellbeing University of Newcastle Newcastle Australia

## Abstract

**Introduction:**

Healthcare is becoming substantially complex in part due to greater multimorbidity, climate‐related health issues, and problems related to access to care. While patient partnership is widely advocated as a strategy to adapt medical practice to the complexity, significant barriers persist.

**Aim:**

We sought to shed light on the ontological and epistemological ‘tensions' generated by the implementation, sometimes by imposition, of the concept of physician–patient partnership in medical practice.

**Methods:**

This reconceptualization of the distinctive ways of knowing is based on illustrative physician‐patient interactions used to contrast the fundamental belief‐systems that generate ontological and epistemological tensions impeding physician‐patient partnership. We contrast knowledge valued by the prevailing positivist paradigm with the kinds of knowledge patients can contribute to ensure optimal healthcare outcomes.

**Findings:**

Identifying the complementarity of evidence‐based and experiential knowledges leads to a paradigmatic shift in how we perceive, interpret, and use data in the clinical setting. The two co‐existing but distinct ontologies and epistemologies—neither is inherently superior to the other—are essential for a broader and concrete understanding of illness, its experience and its management.

**Conclusion:**

This exploration into the complex nature of physician‐patient partnerships provides insights about avenues for strengthening them and making them fulfil their promise to enhance health care access and outcomes for all.

**Patient Contribution:**

The first author is a kidney transplant recipient (2008) and has been actively involved in patient partnership within health research and medical education since 2010. This paper reflects his accumulated insights and observations regarding the barriers that hinder the development of effective partnerships in health care.

Knowledge is a navigation through an ocean of uncertainty, where one can resupply on islands or archipelagos of certainty.

Edgar Morin [1].

## 1 Introduction

The number of patients presenting complex health problems is poised to increase in part due to increased prevalence of chronic multimorbidity [2, 3], climate‐related health issues [4], and the growing complexity in access and equity in health care [5]. The concept of physician‐patient partnership has been promoted as a means to enhance health outcomes through greater patient involvement [6, 7], yet its implementation in healthcare services continues to be arduous. A persistent obstacle hinted at by recent work [8] is the ontological (what is considered true knowledge) and epistemological (what is considered reliable knowledge) gap between the knowledge physicians and patients bring to the partnership [9]. We argue that for meaningful patient participation in clinical decision‐making, especially in cases where a clear diagnosis is elusive, recognising and managing this gap might advance physician‐patient partnership.

This paper focuses on the ontological and epistemological ‘tensions' generated by the implementation, sometimes by imposition, of the concept of physician‐patient partnership in medical practice. The COVID‐19 pandemic has exacerbated pervasive challenges to physician‐patient partnerships [10, 11]. The disruptions during the pandemic regarding in‐person meetings, and the subsequent growth of tele‐health service delivery, exert pressure on physician‐patient interaction to limit discussion to essential issues. These effects make a reconceptualisation of the distinctive ways of knowing and how they relate to each other particularly timely.

To illustrate the imminent ‘messy' reality of clinical practice, consider Michael (see details in Box 1), who experienced a range of persistent symptoms and clinical signs. Despite the dedicated efforts of multiple clinicians, his condition defied classification under any known diagnosis, leading to both ontological questions and epistemic uncertainties about what can be known.

Box 1. **The Un‐knowable Patient.**

Michael has been unwell for many years, with fluctuating symptoms of fever, fatigue, joint pains, that fit into a dozen well‐documented conditions, yet each test returned ambiguous, contradictory results. Rheumatologists, infectious disease specialists, and neurologists suggested theoretical explanations of his condition, but all crumbled under scrutiny.

The problem wasn't just that Michael's illness was difficult to diagnose, rather it was unclear whether his experiences in fact were caused by a singular disease. His symptoms fluctuated, appearing and vanishing in ways that defied biomedical logic. It was as though the disease itself was unstable, shifting with every new information gathered from Michael's experiences and the resulting medical investigations or interventions.

Both, Michael and his doctors were caught in a maze of ontological (the uncertainty about what exists or what is real) and epistemic uncertainty (about what we can know).

Michael, ontologically, wondered if his illness was real in the way other diseases were real, or if it was something else entirely. He felt like a ghost in his own body, unsure whether his suffering was rooted in something tangible or a failure of language and classification. Was he sick in a way that medicine could eventually define, or did his illness exist in a liminal space, beyond the grasp of science? The shifting nature of his symptoms made him question not just what was wrong with him, but whether an answer was even possible.

His doctors equally found themselves caught in a maze of ontological uncertainty, namely their fear that medicine itself might not fully capture the ‘reality of disease’. They contemplated if the boundaries between illnesses and disease were far less rigid than the textbooks suggested, or if Michael's illness experiences were not one disease but many overlapping conditions, or even worse, something beyond their current medical understandings?

Michael created his own understandings. If those who are supposed to know were uncertain, how could he make sense out of his own bodily experiences? Were they even legible, or could he even trust them when they defied medical logic? At times, he found solace in uncertainty; if no one could pinpoint his illness, then maybe he was not doomed to a singular fate. At other times, knowing that he had no definitive disease erased his suffering.

Recognising the uncertainties of Michael's illness changed his doctors' approaches—instead of searching for a single diagnosis, they examined and treated his condition from a dynamic rather than static perspective. They realised that they could manage the inherent uncertainties of the unknown.

Over time Michael and his doctors learned that medicine was not just about solving mysteries, but about standing with patients when the mysteries remained. For Michael it is the story of adapting to live with probabilities rather than certainties, while for his doctors, it is a story of humility—medicine not always is about finding absolute answers, but the commitment to stand with the patient in the face of the unknown.

To move forward, Michael and his doctors had to shift their focus from finding a definitive diagnosis to actively managing his condition as it evolved. Open sharing of the subjective and objective dimensions of his lived experience allowed the bridging of the ontological and epistemological gap in ‘knowing' between Michael and his doctors. The emerging deeper mutual understanding strengthened the partnership, eased the clinicians' fears of professional inadequacy and helped Michael accept and live with the uncertainty of his undiagnosed condition.

## 2 The Ontological and Epistemological Divide

Physicians are taught to base clinical decisions on knowledge produced through replicable scientific methods embedded in the positivist paradigm. Within this paradigm, knowledge's relevance is determined by the intensity of interactions among observations. To minimise the risk of bias, observations are expressed in unambiguous terms, often in scalar or non‐scalar values. Within this paradigm therefore, expressing knowledge in minimalistic expressions makes ‘evidence' more reassuring to make sound clinical decisions [12].

Consequently, scientific research within this paradigm seeks to establish generalisability of an insight by testing the strength of the connections underpinning it. In real life, this implies that a sustained change in a biomarker on a number, deemed statistically significant, of patients, can be relied on to make clinical decisions for ALL patients. Whereas statistical validation provides probabilistic reliability to confirm drug effectiveness, the tenuousness of this assumption is that what applies to groups of patients should also apply to individuals. As illustrated in Michael's case above, this may be deemed insufficient to apprehend the deeper complexity of a unique health problem [13]. The issue is further conflated when considering what Varela [14] and Kahneman [15] have argued: Humans are notoriously unreliable when it comes to rational, deductive thinking, implying that rationality has a feeble grip on our behaviours [16, 17]. A cursory look at the state of the world is enough to reveal the inadequacy of human ‘reason’ and assorted technological advancements to understand humanity's problems [1].

We argue that the dominant positivist paradigm in healthcare, giving precedence to evidence‐based knowledge, tends to marginalise other forms of knowing, particularly knowledge derived from personal experience [18]. As an illustration of how the different ontological and epistemological understandings of knowledge can hinder partnership development, consider Mrs. Ferguson, who engages with the triage nurse at the Emergency Department (adapted from Deschenes et al. [19]):

Patient (Mrs. Ferguson)—*Since yesterday, I feel I am out of breath, even when I sit on my chair. I hear popping noises in my chest*.

Triage Nurse's note as conveyed to the attending physician—*Mrs. Ferguson is a 51‐year‐old woman who has been experiencing difficulty breathing for the past 24 h at rest and without physical effort. Sounds resembling bubbles popping are heard on auscultation and are localised in both pulmonary bases*.

The attending physician translates this as—*Middle‐aged female presents with an onset of dyspnea at rest and on exertion (e.g., talking) characterised by bilateral crepitation in pulmonary bases on auscultation. Differential diagnoses: congestive cardiac failure (CCF), pneumonia*.

The patient's knowledge, derived from the subjective experience of panic at not breathing, is transformed into the emotionless standardised medical reasoning language [19, 20]:

- ‘Breathlessness’ becomes ‘dyspnea'; effectively evacuating the patient's distress.
- ‘Bubbles' become ‘crepitations' neutralising the subjective quality of experience.

To include Mrs. Ferguson, the person WITH the illness, as a partner in clinical decision‐making, the physician's reasoning needs to acknowledge her experiential knowledge. If not, Mrs. Ferguson is excluded from the conversation. Patients frequently report feeling unseen and unheard in their interactions with healthcare providers, perceiving that they are regarded only in terms of their disease rather than as whole individuals [21]. This erodes trust and hinders the development of meaningful physician–patient relationships.

This example of ‘elaborated knowledge' [20] or the process of articulating clinical data for clinical reasoning [22] illustrates what medical students are trained to do. Instead of being trained to enrich their clinical reasoning by including patient's experiential knowledge, they are trained to reduce their reasoning to common denominators. This effectively sidelines patient knowledge considered insufficiently relevant for clinical reasoning, what Dotson labels as epistemic oppression [23].

The belief that knowledge must be generalisable and demonstrable hinders attempts to consider patients lived experiences as valid contributions to clinical decision‐making [24]. Alarmingly, the belief reinforces the illusion that clinical decision‐making is appropriate only when it leads to ‘one correct answer,’ [25] as reflected in the conundrum Michael's doctors were in.

## 3 Embracing Patients' Ways of Knowing—Bridging the Gap

To begin the exploration as to how this knowledge could be integrated into clinical reasoning, it is important to describe the paradigm in which patients construct their knowledge, namely, what this knowledge considers true, its ontology, and how patients come to rely on it, its epistemology.

The way patients develop their experiential knowledge is ascribed to the pragmatic paradigm, coherent with John Dewey's views on learning from experience [26]. Experiential learning is rooted in the process of examining and testing beliefs and personal theories to generate new and more refined ideas [27]. This process is continuous and unique—everyone enacts it differently—and ‘what [an individual] has learned in the way of knowledge and skill in one situation becomes an instrument of understanding and dealing effectively with the situations which follow’ (p. 35) [26]. Hence, within this perspective, experience is considered ontologically true and epistemologically reliable when the meaning made from it is shared within a community familiar with such experiences [28, 29, 30, 31].

A crucial implication within this paradigm, is that knowledge cannot be considered definitive, it is permanently ‘under construction.' [32] Although this might appear in stark contrast to the positivist paradigm, where knowledge is considered immutable and its relevance hinges on its replicability, Polanyi's post‐critical philosophy, provides a way to bridge the gap [33, 34], by suggesting that any knowledge humans hold about the world is necessarily embedded within a man‐made structure; an imposed theoretical order on the randomness of nature [33].

Therefore, the knowledge that patients gain from direct experiences with their bodies through interoception [35] and interactions within their environment [36] is considered legitimate within the pragmatic paradigm. Patient knowledge about internal body states leads to a heightened awareness of their health needs, preferences, and what feels right and wrong, and ultimately guides their behaviour and decision‐making [37, 38]. It is this knowledge, increasingly emphasised in recent literature about patient involvement in healthcare, that physicians need to learn to integrate into their clinical decision‐making.

Authors have pointed to embodied knowledge—akin to Polanyi's concept of tacit knowledge—embedded in bodily processes which unconsciously, yet effectively, guides patients' personal decision‐making [39, 40]. Patients inevitably ‘monitor' their physical and psychological state, and when they can no longer ‘make sense' of their state, they seek to expand their knowledge base. The interaction with physicians allows them to gain complementary knowledge about physiological causes and mechanisms generating illness, their symptoms, their treatment options, and the potentials of drug interactions. This ‘medical' knowledge adds to what can be considered patient knowledge [9].

In addition, patients develop ‘relational' knowledge [9] about who to rely on within their community, how to access needed care, and who can help them with disease management to support their life goals. Increasing relational knowledge helps to engage with clinicians by learning to use medical language to advocate for themselves. Through experience, they also acquire what Dumez et al. call ‘navigational' knowledge which helps them effectively access appropriate healthcare and social support services. And lastly, ‘cultural' knowledge allows patients to identify how norms and values shape their care preferences and expectations in their health journey. Table 1 presents the wealth and depth of patient knowledge available for clinical decision‐making

**Table 1:** The richness of patient knowledge [9, 37].

Patients develop various types of knowledge over time:
Embodied Knowledge: Patients gain an intuitive understanding of their bodies' signals through lived experience. Relational Knowledge: Patients learn how to navigate healthcare systems and build relationships with providers. Cultural Knowledge: Patients understand how cultural norms influence health perceptions and care preferences. Medical Knowledge: Through interactions with healthcare professionals, patients acquire technical insights about their conditions. Navigational Knowledge: Patients become adept at accessing resources within healthcare systems.

To illustrate how patient knowledge can orient clinical decision making, consider a patient telling the physician they refuse a treatment option because, from experience, they know the drug's side effect will exacerbate unpleasant symptoms (embodied knowledge); or a patient becomes skillful in reading the physician's level of fatigue and picks the correct moment to ask difficult questions (relational knowledge), or; a patient considers that a proposed therapy will be severely diminish their body energy levels (medical knowledge).

Unpacking a patient's experience to apprehend its meaning becomes a valuable epistemic exercise that leads to expanded knowledge about disease and its management [41]. These diverse forms of knowledge (see Table 1) empower patients to engage meaningfully in clinical decision‐making.

## 4 How to Optimise the Complementarity Among Knowledges

Despite widespread efforts to implement physician‐patient partnership in view of enhancing healthcare outcomes [42], the ontological and epistemological tensions, as outlined above, persist as a formidable obstacle [43, 44, 45]. To move forward and attenuate these tensions, it is necessary to acknowledge the complementarity of biomedical and experiential knowledge [46] to optimise relevant knowledge for clinical decision‐making [18, 47, 48]. Table 2 presents a comparative analysis of both types of knowledge that could form the basis for eliciting the complementarity between them.

**Table 2:** Comparing biomedical and experiential knowledge.

	Biomedical Knowledge	Experiential Knowledge
Characteristics	Derived from population‐level studies Focused on pathology Focused on objective measures and biomarkers Validated through peer‐reviewed research	Derived from the experience of personal illness Focused on impacts on quality of life Embedded in social and environmental contexts Validated pragmatically through bodily awareness and observable outcomes
Strengths	Reliability Generalisability Reproducibility Focus on the effects between variables	Depth of understanding Descriptive richness Credibility Applicability
Limitations	Superficial information Excludes significant but subtle individual variations Low descriptive power	High interdependence Narrow insights Limited scope
Clinical Relevance	Acute emergencies Diagnostic certainty Evidence‐based treatments	Chronic disease management Quality‐of‐life decisions Treatment adherence

Table 2 illustrates possible complementarities between biomedical and experiential knowledge, which echo complementarities between quantitative and qualitative data in research. Both seek to understand human adaptation to changing circumstances but pursue different purposes. Biomedical knowledge comprises confirmation of hypothesised interactions about known entities (molecules, organic reactions, homoeostasis, etc.) whilst experiential knowledge comprises descriptions of unitary observations shared within a community. Both seek to highlight ways to ensure patient well‐being. The respective strengths and limitations indicate how they compensate for what the other doesn't have.

To conceptualise, we refer to the field of genealogy, based on Foucauldian philosophy, which is posited as an alternative to ‘unitary sciences'. Unitary sciences focus on common patterns within large data sets, whereas genealogy focuses on data that does not fit common patterns, expressed by genealogists as ‘local knowledge'. [13] In essence, genealogy emphasises the concrete, contextual character of knowledge and seeks to unravel interactions underneath common patterns of data [49]. Finally, as suggested by Jørgensen [50], genealogy recognises the importance for knowledges to preserve their distinct nature while contributing equally to deeper understanding of a given phenomenon.

Patient‐reported outcome measures (PROMs) and Patient Reported Experience Measures (PREMs) reflect the importance health‐care professionals and service administrators attribute to patients' preferences. The primary aim of these measures, firmly embedded in a unitary sciences approach, is to leverage knowledge of patient preferences to enhance the value of health care as well as system performance. The degree to which patients contribute their experiential knowledge in developing these measures varies however and has garnered some attention [51, 52]. In fact, Wiering et al. found that preferences may differ widely between patients, implying important limitations in seeing patient reported measures as a way to combine knowledges to enhance physician‐patient partnership.

Complementarity of physician and patient knowledge is at the core of shared decision‐making (SDM) as recently described by Montori et al. [53] and Thomas [18]. SDM is described as an iterative and continuous process where the physician and patient carefully listen to each other until a response emerges that: (1) is evidence‐based and relevant to the situation; (2) is feasible and minimally disruptive of personal and social routine, and (3); accounts for the emotional dimensions of the situation, ensuring both patient and physician feel “it is the right thing to do now” [53] (p. 213). Work by Bilodeau et al. [30, 41, 54] on parallel care journeys of clinicians and patients revealed spaces of complementarity whose effects work to enhance healthcare experiences and outcomes. Finally, the row on Table 2 concerning clinical relevance suggests that both types of knowledge sit on the same continuum between initial onset of symptoms requiring acute care where the patient's involvement is limited, to chronic care, which is heavily dependent on patient involvement, hence the greater relevance of experiential knowledge.

## 5 Discussion

Medical practice must evolve as an adaptive system [55] to address complex, novel situations in a perpetually evolving environment, namely by integrating experiential knowledge into medical training [56]. Adaptive expertise comprises learning to expand the knowledge base to select from and apply to clinical‐decision making which will strengthen physician‐patient partnerships as well as enhance healthcare outcomes. This calls for an exploration of ways to integrate knowledge grounded in separate ontological and epistemological understandings.

One limitation of the cognitive model of clinical reasoning is its implied linearity, in which clinical signs must be matched to pre‐existing diagnostic patterns. This approach is inherently reductionist, as it prioritises knowing over understanding and reinforces a mechanistic view of medical diagnosis. This disparity is perpetuated across generations of trainees due to the emphasis placed on teaching clinical reasoning as a matching process that leads to a single correct answer. However, given the increasing complexity of healthcare, effective clinical reasoning [57] demands the ability to listen actively and engage holistically with the patient‐perceiving dimensions of their experience that may not align neatly with an established, match‐based cognitive process.

**Figure 1:**
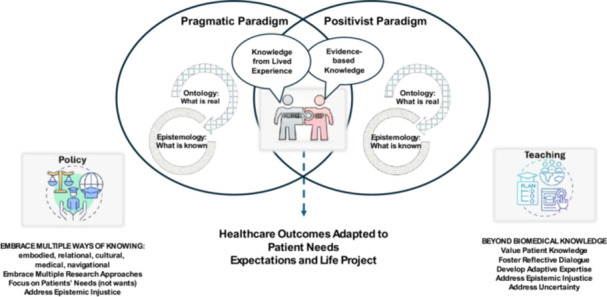
Fostering clinician–patient partnerships.

We argue that the integration of evidence‐based and experiential knowledges for clinical decision‐making calls for a paradigmatic shift in how we perceive, interpret, and use data in the clinical setting [6]. Both paradigms reflect two co‐existing but distinct ontologies and epistemologies—neither is inherently superior to the other [58]; however, both are essential for a holistic understanding of illness and management of care [43, 59]. Figure 1 illustrates the intersection between the ontological and epistemological understandings and its implication for teaching, that we have argued could bridge the gap.

We have argued that to foster physician–patient partnerships, it is important to embrace the differences on the ontological and epistemological level, not erase them. Past efforts to equate patient experiential knowledge with evidence‐based knowledge failed because they ignored ontological and epistemological tensions. Treating *the knowledge of the ‘expert patient'* as a form of medical information dilutes its *unique value* [60] and is another form of reductionism, and thereby perpetuates *the epistemic injustice that proponents were trying to counter* [60] (p. 3–4). Instead of blending knowledge types, we posit a coexistence model that honors their complementarity: experiential knowledge helps reveal blind spots in scientific reasoning and vice versa [61, 62, 63].

### 5.1 Knowledge Integration Through Dialogue

These ontological and epistemological considerations imply a hierarchical tension between these two types of knowledge [64] and a struggle against injustice and oppression [65]. We argue that recognising the tensions highlights the need to build capacity to meaningfully integrate patients' experiential knowledge into clinical care [8, 18]. Such capacity‐building, strengthening adaptive expertise, has been integrated in programs that include patient voices in interprofessional education [66] and purposefully narrowing the gap between the learning context and real‐world practice [67, 68]. Yet, efforts to integrate patient partnerships holistically have been very limited, as exemplified by limited scope initiatives on empathy training [21] and compassion training [69]. Greater efforts have been devoted to building researcher capacity to integrate patients as partners in what is known as patient‐oriented research (POR) [70]. Yet they have fallen short of producing deeper insights into how experiential knowledge can be invested. Instead, the focus has been on promoting the concept of patient‐partnership by extolling its virtues to a presumably sceptic audience and presenting exemplary success stories [71, 72], or showcasing valuable health outcomes and tools [73, 74]. Other groups have approached patient‐partnership as a teamwork development issue [75, 76], a role attribution issue [77] or as a structural political issue generating inequalities [10, 49, 78]. Few authors have addressed the deeper ontological and epistemological tensions with a view to removing the obstacles to combining multiple knowledges [56, 59, 60].

Consequently, among the myriad efforts to train physicians to communicate effectively TO patients [79, 80, 81], there are few, if any, initiatives where trainees and patients practice what some authors call ‘reflexive dialogue' [82] WITH patients. The need to foster a common vocabulary and language patterns through dialogue, aligned with social‐constructivist views of learning, to support learning about complexity and uncertainty, is slowly gaining ground in medical education [83, 84, 85]. From a learning perspective, the importance of meaning making from experience as a means to comprehend complex phenomena [86] has been demonstrated convincingly for close to a century now [26, 87, 88].

### 5.2 Refocusing on Patient Care and Shared Decision‐Making

Physician–patient partnership also requires a framework that values and rewards adaptive expertise [89], allowing clinicians to continually evaluate experiential and biomedical knowledge so that they can meet unique patients' needs in specific contexts. This will require medical training to acknowledge diverse forms of knowledge by demonstrating greater ontological flexibility when applying evidence‐based protocols (Table 3). Cognitive apprenticeship‐based teaching models [90], where instructors deliberately make their expert clinical reasoning visible to learners, have been shown to effectively cultivate sensitivity to complexity and reinforce the integration of all available knowledge [91] for clinical reasoning. While much research has been conducted on the relevance of the cognitive‐apprenticeship model to health sciences education, very little attention has been devoted to its ontological and epistemological avenues when patients co‐teach or engage in reflexive dialogue with students.

**Table 3:** Key considerations for bedside teaching.

Teach how to embed patient knowledge into clinical reasoning. Value patient knowledge: Recognise and legitimize the diverse forms of knowledge patients bring to healthcare. Foster reflective dialogue: Encourage physicians to engage in reflective practice and dialogue with patients. Develop adaptive expertise: Shift from rigid protocols to flexible, context‐sensitive approaches. Address epistemic injustice: Challenge the hierarchical privileging of scientific knowledge over experiential knowledge.

## 6 Conclusion

Experiential knowledge held by patients about their body is required to form the adaptive and contextualised health plans that lead to better health outcomes. Dismissing this knowledge leads to the marginalisation of patients' voice in clinical decision‐making; effectively excluding patients from meaningful participation in their own care. The COVID‐19 pandemic has further highlighted the urgency of addressing these tensions, as the need for collaborative, person‐centered care becomes ever more apparent.

Bridging this divide requires more than simply acknowledging different knowledge types; it demands a deliberate effort to foster dialogue, develop a shared language, and integrate both biomedical and experiential knowledge into clinical practice. Unfortunately, at present, the pragmatic paradigm's view on what is true and reliable knowledge, does not enjoy equal status as the positivist paradigm. The leveling‐of‐the‐field we advocate for will enable co‐creation of care plans that are both evidence‐informed and responsive to individual needs, ultimately strengthening physician‐patient partnerships and improving health outcomes.

## Author Contributions

**Nicolas Fernandez:** study design, literature review, patient perspective and writing. **Joachim P. Sturmberg:** study design, medical practitionner perspective and review of manuscript drafts.

## Ethics Statement

The authors have nothing to report.

## Conflicts of Interest

The authors declare no conflicts of interest.

## Data Availability

The authors have nothing to report.
